# Simultaneous quantitation of four androgens and 17‐hydroxyprogesterone in polycystic ovarian syndrome patients by LC‐MS/MS

**DOI:** 10.1002/jcla.23539

**Published:** 2020-08-21

**Authors:** Zheng Cao, Yifan Lu, Yuting Cong, Ying Liu, Youran Li, Husheng Wang, Qiaoli Zhang, Wenxi Huang, Jingrui Liu, Ying Dong, Guodong Tang, Yiqi R. Luo, Chenghong Yin, Yanhong Zhai

**Affiliations:** ^1^ Department of Laboratory Medicine Beijing Obstetrics and Gynecology Hospital Capital Medical University Beijing China; ^2^ Shanghai AB Sciex Analytical Instrument Trading Co., Ltd. Shanghai China; ^3^ Department of Obstetrics Beijing Obstetrics and Gynecology Hospital Capital Medical University Beijing China; ^4^ Department of Human Reproductive Medicine Beijing Obstetrics and Gynecology Hospital Capital Medical University Beijing China; ^5^ Guangzhou Kangrun Biotech Co., Ltd Guangzhou China; ^6^ Department of Laboratory Medicine University of California San Francisco San Francisco CA USA; ^7^ Central Laboratory Beijing Obstetrics and Gynecology Hospital Capital Medical University Beijing China

**Keywords:** androgen, diagnosis, LC‐MS/MS, PCOS, validation

## Abstract

**Background:**

Due to the low concentration of androgens in women and the limitation of immunoassays, it remains a challenge to accurately determine the levels of serum androgens in polycystic ovary syndrome (PCOS) patients for clinical laboratories. In this report, a liquid chromatography‐tandem mass spectrometry (LC‐MS/MS) method was developed and validated for simultaneous quantitation of testosterone (T), androstenedione (A4), dehydroepiandrosterone sulfate (DHEAS), dihydrotestosterone (DHT), and 17‐hydroxyprogesterone (17‐OHP) that are associated with PCOS.

**Methods:**

The serum samples were processed by protein precipitation and solid phase extraction before analysis with the in‐house developed LC‐MS/MS. The chromatographic separation was achieved with a C18 column, using a linear gradient elution with two mobile phases: 0.02% formic acid in water (phase A) and 0.1% formic acid in methanol (phase B). The separated analytes were detected by positive or negative electrospray ionization mode under multiple reaction monitoring (MRM).

**Results:**

The assay for all the five analytes was linear, stable, with imprecision less than 9% and recoveries within ±10%. The lower limits of quantification were 0.05, 0.05, 5, 0.025, and 0.025 ng/mL for T, A4, DHEAS, DHT, and 17‐OHP, respectively. In the receiver operating characteristic curve (ROC) analyses with the PCOS (n = 63) and healthy (n = 161) subjects, the AUC of the four‐androgen combined was greater than that of any single androgen tested in PCOS diagnosis.

**Conclusions:**

The LC‐MS/MS method for the four androgens and 17‐OHP showed good performance for clinical implementation. More importantly, simultaneous quantitation of the four androgens provided better diagnostic power for PCOS.

## Graphical abstract

An in‐house LC‐MS/MS method was developed and validated for simultaneous quantitation of testosterone (T), androstenedione (A4), dehydroepiandrosterone sulfate (DHEAS), dihydrotestosterone (DHT), and 17‐hydroxyprogesterone (17‐OHP) that are associated with polycystic ovary syndrome (PCOS). With PCOS patients and healthy controls, the combinational detection of the four androgens (T, A4, DHEAS, DHT) was superior to any of the single androgen in PCOS diagnosis. (A: The complete chromatograms of the five analytes resolved by the established LC‐MS/MS method: DHEAS (1), A4 (2), T (3), 17‐OHP (4), and DHT (5). B: The ROC analyses were performed with the 63 PCOS patients and 161 healthy controls. The combination detection referred to the simultaneous measurements of the four androgens of T, A4, DHEAS, DHT).

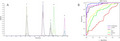

## 1 INTRODUCTION

Polycystic ovary syndrome (PCOS) is recognized as one of the most common endocrine disorders in child‐bearing aged women around the world^1^ and is complicated with reproductive, metabolic, and psychological features.^2^ However, the etiology of this disease remains largely unknown. Because of the heterogeneity in its clinical presentations, the diagnostic criteria of polycystic ovary syndrome have been debatable,^3^ which poses a huge challenge for its clinical diagnosis.^4^ Three significant diagnostic features of PCOS—chronic anovulation, hyperandrogenism, and polycystic ovaries ^1^, ^5^—had been proposed and gained wide acceptance.^1^, ^5^, ^6^

Hyperandrogenism plays a prominent role in the pathological process of PCOS,^7^ and it's considered as the most constant and important diagnostic component of this syndrome.^6^, ^8^, ^9^ However, which androgens should be measured for the diagnosis of PCOS is still controversial. Ideally, the serum levels of testosterone (T), androstenedione (A4), dehydroepiandrosterone sulfate (DHEAS), dihydrotestosterone (DHT), and 17‐hydroxyprogesterone (17‐OHP) were suggested to assess the origins and the extent of excessive androgens of women.^10^, ^11^

On the other hand, direct immunoassays (IAs), such as radioimmunoassay (RIA), chemiluminescence, and enzyme immunoassays, are the most widely used methods for measuring androgens in clinical laboratories.^6^, ^12^ The IAs were found to be susceptible to temperature, pH value ionic strength, and other factors,^13^ resulting in relatively poor sensitivity and specificity.^12^, ^13^ More importantly, IAs are prone to generate erroneous results and overestimate the androgens levels due to the unavoidable antibody‐antigen cross‐reactions. As a result, the IAs have been advised against being applied in the androgen measurement in women and children.^12^ Recently, the liquid chromatography‐tandem mass spectrometry (LC‐MS/MS)‐based methods, with high sensitivity and “gold‐standard” specificity,^14^, ^15^ have been employed to measure steroids in human serum.^14^ Many studies have confirmed that the LC‐MS/MS had good performance in the determination of T, DHEAS, A4 or other steroids,^16^, ^17^, ^18^ offering an ideal alternative methodology in the determination of hyperandrogenism in PCOS.^19^ Some previous studies have shown that the simultaneous determination of T and A4, or T and DHT was achievable with excellent accuracy and little matrix interference.^20^, ^21^ As part of the continual efforts to improve the laboratory diagnostic accuracy of hyperandrogenism in PCOS patients, we established an efficient LC‐MS/MS method for simultaneous measurement of serum T, A4, DHEAS, DHT, and 17‐OHP and evaluated the clinical utility of this androgen testing panel with the PCOS patients.

## 2 MATERIALS AND METHODS

### 2.1 Chemicals and reagents

Methanol (Optima^®^ LC/MS grade), formic acid (LC/MS grade, 98%), and acetonitrile (Optima^®^ LC/MS grade) were purchased from Fisher Scientific. The hormone‐free human plasma (blank plasma, product code:1800‐0058) was purchased from Shanghai Pufeng Biotechnology. All certified reference standards (catalog number, concentration, purity): testosterone (T‐037, 1.0 mg/mL, 99.7%); androstenedione (A‐075, 1.0 mg/mL, 99.7%); dehydroepiandrosterone sulfate (D‐065, 1.0 mg/mL, 99.9%); dihydrotestosterone (D‐073, 1.0 mg/mL, 99.9%); 17‐hydroxyprogesterone (E‐060, 1.0 mg/mL, 99.7%), and isotope‐labeled internal standard (IS) solutions: T‐d3 (T‐046, 100 μg/mL, 99.0%); A4‐^13^C3 (A‐084, 100 μg/mL, 99.8%); DHEAS‐d5 (D‐066, 100 μg/mL, 99.6%); DHT‐d2 (D‐077, 100 μg/mL, 98.1%); 17‐OHP‐^13^C3 (E‐117, 100 μg/mL, 98.1%) were purchased from Cerilliant.

### 2.2 Calibrators, quality control samples, and internal standard solutions

The 10‐fold calibrators and quality control (QC) working solutions were prepared from methanol stock solutions of a concentration of 1.0 mg/mL for all the chemical standards and were stored at −20°C. For daily use, the six‐point calibrators or QCs were prepared by mixing one part of the 10‐fold working solution (5 μL) with 10 parts of the blank plasma (50 μL). As a result, the following equivalent concentrations for the calibrators and the QCs were achieved: six‐point calibrators (T: 0.05, 0.1, 0.2, 0.60, 3, 6 ng/mL, A4: 0.1, 0.2, 0.4, 1.2, 6.0, 12 ng/mL, DHEAS: 10, 20, 40, 120, 600, 1200 ng/mL, DHT: 0.05, 0.10, 0.20, 0.60, 3, 6 ng/mL, 17‐OHP: 0.05, 0.10, 0.20, 0.60, 3, 6 ng/mL); high‐level QC (QC‐H) (4.80 ng/mL T, 9.60 ng/mL A4, 960 ng/mL DHEAS, 4.80 ng/mL DHT, 4.80 ng/mL 17‐OHP), medium‐level QC (QC‐M) (0.60 ng/mL T, 1.20 ng/mL A4, 120 ng/mL DHEAS, 0.60 ng/mL DHT, 0.60 ng/mL 17‐OHP), and low‐level QC (QC‐L) (0.10 ng/mL T, 0.20 ng/mL A4, 20.00 ng/mL DHEAS, 0.10 ng/mL DHT, 0.10 ng/mL 17‐OHP). The IS mixture was prepared with methanol, acetonitrile, and water (1:1:2) at the following concentrations: 10.00 ng/mL T‐d3, 2.00 ng/mL A4‐^13^C3, 5.00 ng/mL DHEAS‐d5, 20.00 ng/mL DHT‐d2, 2.00 ng/mL 17‐OHP‐^13^C3.

### 2.3 Sample preparation

For each sample preparation, 50 μL patient serum + 5 μL methanol or 55 μL plasma‐diluted QC/calibrator, 10 μL internal standard mixture, 145 μL methanol and 150 μL water were mixed in a 96‐well protein precipitation plate. The mixture was shaked for 1 minute. Then, the supernatant was transferred to the Agela Cleanert PEP 96 Well Microplates for solid phase extraction, followed by a washing step with 200 μL of 10% acetonitrile in hexane. Lastly, the analytes of interest were eluted by 50 μL methanol/acetonitrile (1:9, v/v) and collected by a 96‐well collection plate. The flow‐through was further diluted with 50 μL water and 10 μL was injected for the LC‐MS/MS analysis.

### 2.4 Instrumentation and conditions

The LC‐MS/MS analysis was performed using an AB Sciex 5500 mass spectrometer coupled with a Shimadzu Nexera X2 high‐performance liquid chromatography (HPLC) system. A Venusil MP C18 column (VA93050, 3.0 × 50 mm, 3 μm, Agela Technologies) was used and maintained at a constant temperature of 40°C during operation. The mobile phase A was composed of 0.02% formic acid in water, and the mobile phase B was composed of 0.1% formic acid in methanol. The chromatography gradient conditions (%B) were set as follows (for a total run time of 6.5 minutes) with a flow rate of 0.6 mL/min: 55% B for 0‐0.5 minutes, 55%‐75% B for 0.5‐3.0 minutes, 75%‐90% B for 3.0‐3.5 minutes, 90% B for 3.5‐4.5 minutes, 90%‐55% B for 4.5‐4.6 minutes, 55% B for 4.6‐6.5 minutes.

The analytes were detected by the mass spectrometer with scheduled multiple reaction monitoring (MRM) in the positive or negative electrospray ionization mode. The source‐specific parameters were as follows: ion spray voltage, 5.5 kV or −4.5 kV; vaporizer temperature, 550°C; collision gas flow 8 L/min; curtain gas flow, 35 L/min and nebulizer gas flow, 80 L/min.

### 2.5 Assay validation

Based on the recommendations of the Clinical and Laboratory Standards Institute (CLSI) 62‐A guideline,^22^ the method was validated for linearity, lowest limit of quantitation (LLOQ), precision, accuracy, matrix effect, serum sample stability, selectivity, carry over, and product ion cross‐talk as described in the following paragraphs.

### 2.6 Linearity and LLOQ

The linearity was evaluated by measuring the ratio of analyte peak area to the IS area against nominal concentrations of calibrators with linear regression and 1/*X* ^2^ weighing. The linearity validation was performed in three times of the same experimental day and the average slope, intercept, and correlation coefficient R of the three repeats were reported. The acceptance criterion for a calibration curve was a correlation coefficient R of 0.990 or better. The LLOQs were calculated by analyzing the serially diluted QC specimens spiked with IS over 5 days. The LLOQ was defined as the average concentration at which the S/N ratio > 10 and CV < 20% and bias was within ±20%.

### 2.7 Precision, accuracy, stability and matrix effect

The intra‐assay imprecision was estimated by analyzing the QCs for five times in the same run. The inter‐assay imprecision was estimated by analyzing the QCs twice a day for a total of 10 days. The accuracy was evaluated by the recovery studies, in which the recovery of each androgen was calculated at high‐, medium‐, and low‐level QCs by comparing the IS peak area ratio of extracted QC samples to the IS peak area ratio of non‐extracted standard solutions at the same concentration. The stability of the analytes in serum was assessed by evaluating serum samples kept at 4 and 21°C (room temperature) for 6 days. The matrix effect was assessed according to the study by Matuszewski et al.^23^ Briefly, the sample A was basically QC‐L, QC‐M, or QC‐H prepared in 50% methanol in water (as neat sample), and it reflected 100% recovery with no matrix effects. The sample B was essentially the extracted blank plasma or serum pool of 20 healthy women. Then, the sample B extraction was split into two parts: One part was spiked with standard chemicals (prepared in methanol), with the final concentrations equivalent of QC‐L, QC‐M, or QC‐H (sample B1); the other part was spiked with pure methanol (sample B2). With the MS responses of each compound from samples A, B1, and B2, the matrix effect was calculated with the following formula: [(B1 − B2)/A − 1] * 100%.

### 2.8 Selectivity, carry over, and product ion cross‐talk

According to the CLSI 62‐A guideline,^22^ it is important to confirm within the testing system (including sample processing and LC‐MS) that the method has low background noise allowable for the assay. For the selectivity validation, the human hormone‐free plasma with no addition of standards was prepared and analyzed as the double‐blank control with the LC‐MS/MS method.

Carry over was assessed by running a human hormone‐free plasma sample immediately after injecting a calibrator 6 (upper limit of calibration curve) sample to verify the minimal sample carry over. The calculated carry over in the blank sample should be less than 25% of LLOQ to be acceptable.^22^

To check whether any product ion cross‐talk exists for the analytes that have identical product ions,^22^ we simply monitored all the ion transitions pairs listed in Table 1 at the chromatographic retention time of each analyte of interest (T, A4, DHEAS, and 17‐OHP sharing the primary product ions with the *m/z* of 97.0).

**TABLE 1:** The analytical and instrumental parameters of the LC‐MS/MS method

Analytes	Q1^a^, *m/z*	Product ions	Q3^b^, *m/z*	DP, V	CE, V	CXP, V
T	289.3	Quantifier	97.0	160	30	11
		Qualifier	109.0	160	34	11
T‐C3	292.3	IS	97.1	160	31	10
A4	287.3	Quantifier	97.0	170	31	11
		Qualifier	109.0	170	31	11
A4‐C3	290.3	IS	100.1	165	30	12
DHEAS	367.1	Quantifier	97.0	‐30	‐20	‐8
		Qualifier	80.0	‐30	‐30	‐10
DHEAS‐D5	372.1	IS	98.0	‐170	‐45	‐8
DHT	291.3	Quantifier	255.3	170	22	9
		Qualifier	159.1	170	32	13
DHT‐C3	294.3	IS	258.3	190	22	13
17‐OHP	331.3	Quantifier	97.1	170	31	11
		Qualifier	109.1	170	35	11
17‐OHP‐C3	334.4	IS	112.0	130	47	11

Abbreviations: CE, collision energy; CXP, collision cell exit potential; DP, declustering potential; IS, internal standard.

^a^ Q1, parent ion.

^b^ Q3, product ions including quantifier and qualifier.

### 2.9 Patients for PCOS evaluation and reference interval studies

Totally, 63 untreated PCOS patients^2^ visiting the Endocrinology Department and 161 healthy females that were seeking for pre‐pregnancy checkups at the Internal Medicine Department were enrolled, from January to August of 2019. The ethical approval from the Beijing Obstetrics and Gynecology Hospital Research Ethics Committee and written informed consents from patients were obtained. The serum samples were collected from the above recruited subjects who were at their menstruation period of day 2‐5. This blood sampling window was recommended for the sex hormones including androgens measurements, according to the PCOS clinical practice guidance implemented at our hospital.

The reference intervals (RIs) for the four androgens and 17‐OHP were derived from the 161 healthy women, with the nonparametric approach according to the Clinical and Laboratory Standards Institute (CLSI) guideline EP28‐A3C.^24^ The receiver operating curve (ROC) analyses were performed with the Sigmaplot 14.0 (Systat). The area under the curve (AUC) represents the discriminating power between the PCOS patients and healthy controls, with *P* values < .05 considered statistical significance. The integrated discrimination improvement (IDI) indices were calculated according to the literature^25^ using a customized Visual Basic for Applications (VBA) program in Microsoft Excel software.

## 3 RESULTS

### 3.1 LC‐MS/MS method optimization

**FIGURE 1:**
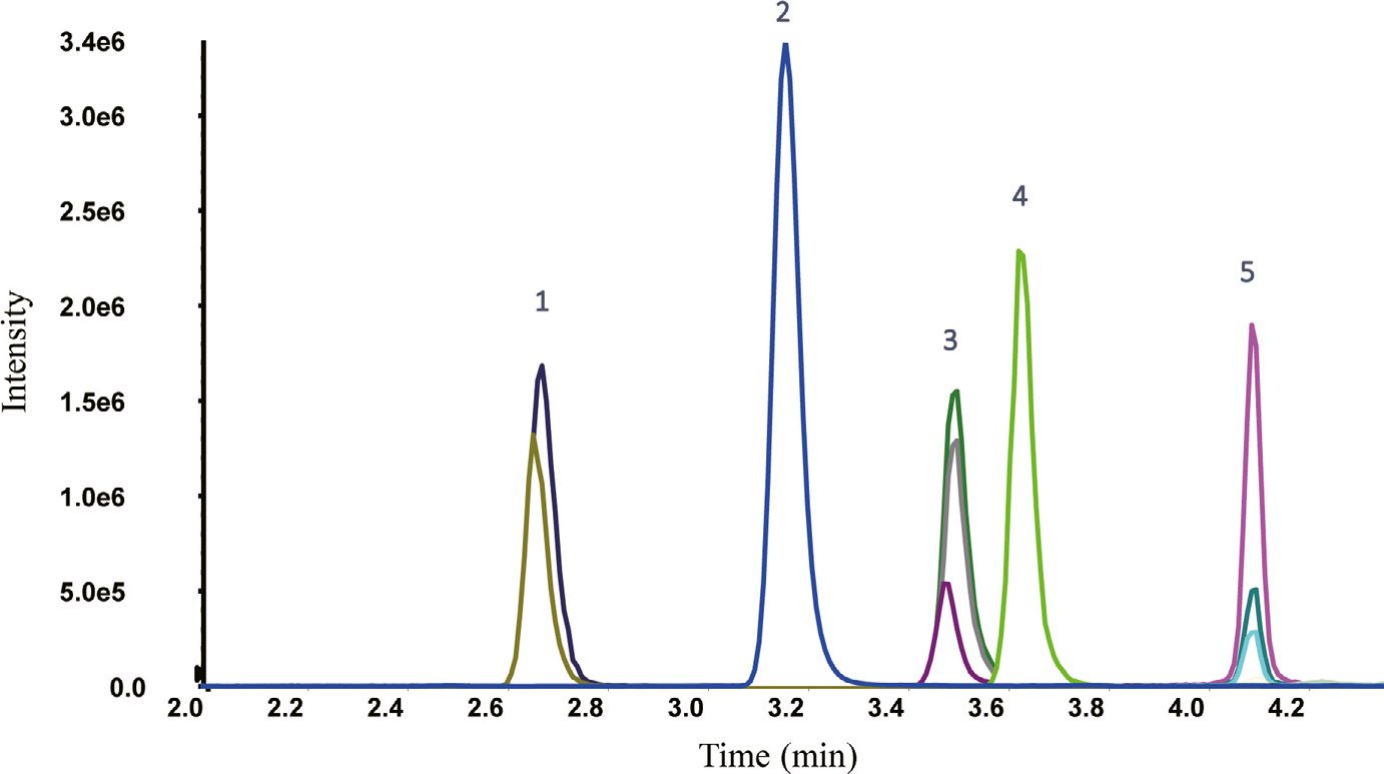
The complete chromatograms of the five analytes resolved by the established LC‐MS/MS method. The peak labels were as follows: DHEAS (1), A4 (2), T (3), 17‐OHP (4), and DHT (5). There were three peaks representing quantifier ions, qualifier ions, and internal standards separately under each compound in the chromatogram

The mass spectrometry instrumentation and conditions, including cone energy voltages, MRM transitions (including parent and product ions of quantifier, qualifier, and internal standard for each analyte), and collision energy voltages, were optimized for each analyte, and the values were listed in Table 1. The serum concentrations of androgens are low in female, making the sample processing a crucial step to warrant the accuracy of the method with acceptable detection limits. The combinational application of protein precipitation and solid phase extraction applied in our method was approved to be a valid and efficient way of extracting a trace amount of androgens in women's blood. Together with the mobile phases and chromatography settings mentioned in the Methods section, the chromatogram of the well‐resolved peaks for T, A4, DHEAS, DHT, and 17‐OHP was shown in Figure 1, with the retention times of 3.49, 3.15, 2.47, 4.08, and 3.63 minutes, respectively.

### 3.2 Assay validation summary

The linearity of the assay was tested by regression analysis, and the correlation coefficient *R* values for all five analytes were >.995. The slopes and intercepts of the linear regression equations for each analyte were listed in Table 2. The linear ranges of the method were as follows: 0.05‐6 ng/mL for T, 0.1‐12 ng/mL for A4, 10‐1200 ng/mL for DHEAS, 0.05‐6 ng/mL for DHT, and 0.05‐6 ng/mL for 17‐OHP. The LLOQs were determined as 0.05, 0.05, 5.0, 0.025, and 0.025 ng/mL for T, A4, DHEAS, DHT, and 17‐OHP, respectively (Table 2). The 95% confidence intervals (CIs) of linearity, precision, and sensitivity studies were provided in the Tables S2 and S3. The intra‐assay CV for QC‐L ranged 5.2%‐8.7%, for QC‐M ranged 3.2%‐5.0%, and for QC‐H ranged 3.7%‐6.3%; the inter‐assay CV for QC‐L ranged 6.5%‐8.2%, for QC‐M ranged 4.2%‐6.0%, and for QC‐H ranged 4.1%‐5.9%. The accuracy for each analyte was evaluated by the recovery studies as described in the Methods. The accuracy measured by % bias was within ±20% acceptance criteria at QC‐L, QC‐M, and QC‐H levels from intra‐ or inter‐assay experiments (Table 3 and Table S3). The analytes were found to be stable in serum for at least 6 days, when stored at 4 or 21°C (Table 2).

**TABLE 2:** The linearity, LLOQ, and stability in assay validation

Analytes	*R*	Linear regression		LLOQ (ng/mL)	Stability, %	
		Slope (±SD)	Intercept (±SD)		4°C^a^	21°C^a^
T	0.9965	0.569 ± 0.030	0.005 ± 0.006	0.05	117.1	104.6
A4	0.9990	0.196 ± 0.009	0.008 ± 0.007	0.05	104.0	110.0
DHEAS	0.9994	0.0012 ± 0.001	0.0032 ± 0.0032	5.0	93.0	92.6
DHT	0.9981	1.154 ± 0.049	0.015 ± 0.016	0.025	101.3	107.0
17‐OHP	0.9988	0.076 ± 0.003	0.003 ± 0.003	0.025	105.0	100.5

Abbreviation: SD, standard deviation.

^a^ Recoveries for serum samples stored at 4 or 21°C for 6 d.

**TABLE 3:** The recoveries, matrix effects, and imprecisions in assay validation

	T	A4	DHEAS	DHT	17‐OHP
Recovery, %					
QC‐L	103.0	97.5	99.3	101.0	96.0
QC‐M	104.5	103.7	109.5	103.2	109.3
QC‐H	97.6	98.9	105.4	99.7	102.2
Matrix effect^a^, %					
QC‐L	92.6	118.4	114.9	115.2	120.0
QC‐M	99.4	116.2	112.0	110.0	118.4
QC‐H	107.2	116.3	116.7	117.2	115.3
Matrix effect^b^, %					
QC‐H	112.1	104.3	115.0	106.5	111.6
Intra‐assay CV, %					
QC‐L	8.0	8.5	5.2	8.7	5.4
QC‐M	5.0	4.1	4.1	3.8	3.2
QC‐H	4.1	4.8	5.1	6.3	3.7
Inter‐assay CV, %					
QC‐L	6.8	8.1	6.5	8.2	7.6
QC‐M	5.7	4.2	5.7	6.0	6.0
QC‐H	5.7	5.1	5.9	5.3	4.1

^a^ Matrix effect with blank serum.

^b^ Matrix effect with serum pool of healthy women.

In the matrix effect assessment (Table 3), the matrix effects with blank plasma or patient serum pool were within 100 ± 20%, showing insignificant ion suppression or signal enhancement. More specifically, the matrix effects with QC‐H were 104.3%‐117.2% in blank plasma and healthy women serum pool; the matrix effects with QC‐L were 92.6%‐120.0%, with QC‐M were 99.4%‐118.4% in blank plasma and were not evaluated with healthy women serum pool due to relatively high background of endogenous hormones.

As seen in Figure S1, the signal from a double blank sample (no analyte, no IS) reflected low background noise in the LC‐MS system. Moreover, the carry over for all the five analytes were acceptable (<25% LLOQ in the blank sample) (data not shown). According to the CLSI C62‐A,^22^ product ion cross‐talk is only of real concern when compounds enter the mass spectrometer at the same time. In Figure 1, no essential peak “overlaps” were observed between any of the analytes of interest. Nevertheless, we still performed the product ion cross‐talk by monitoring all the ion transitions listed in Table 1. No product ion cross‐talk was seen across the entire chromatographic running time (data not shown).

### 3.3 Reference intervals and PCOS evaluation

The reference intervals determined with the healthy controls for T, A4, DHEAS, DHT, and 17‐OHP were 0.08‐0.86, 0.32‐2.33, 273‐2500, ≤0.41, and ≤2.7 ng/mL, respectively (2.5th‐97.5th for two‐sided RIs, and ≤95th for one‐sided RIs) (Table 4). Interestingly, with the upper RI limits as cutoff values, 47.6% (30/63) of PCOS patients had single T elevated; 22.2% (14/63) had T plus at least one of the other three androgens elevated; 6.3% (4/63) showed non‐T (A4, DHEAS or DHT) androgen increase. This result suggested that a portion of PCOS patients presented with only non‐T androgen(s) increase and that the potential clinical utility of simultaneous quantitation of the four androgens was high in PCOS diagnosis.

**TABLE 4:** The reference intervals establishments and the ROC analyses

Analytes	T	A4	DHEAS	DHT	17‐OHP	T + DHT	Combination^a^
Reference intervals, ng/mL	0.08‐0.86	0.32‐2.33	273‐2500	≤0.41	≤2.7		
AUCs in the ROC analyses	0.94	0.52	0.73	0.81	ND^b^	0.95	0.99
95% confidence interval of AUCs	0.90‐0.97	0.44‐0.60	0.66‐0.81	0.74‐0.87	ND^b^	0.92‐0.98	0.97‐1.00
Sensitivity	0.86	0.84	0.76	0.76	ND^b^	0.89	0.95
Specificity	0.90	0.25	0.65	0.70	ND^b^	0.94	0.96

^a^ Combination detection of T, A4, DHEAS and DHT.

^b^ Not determined.

**FIGURE 2:**
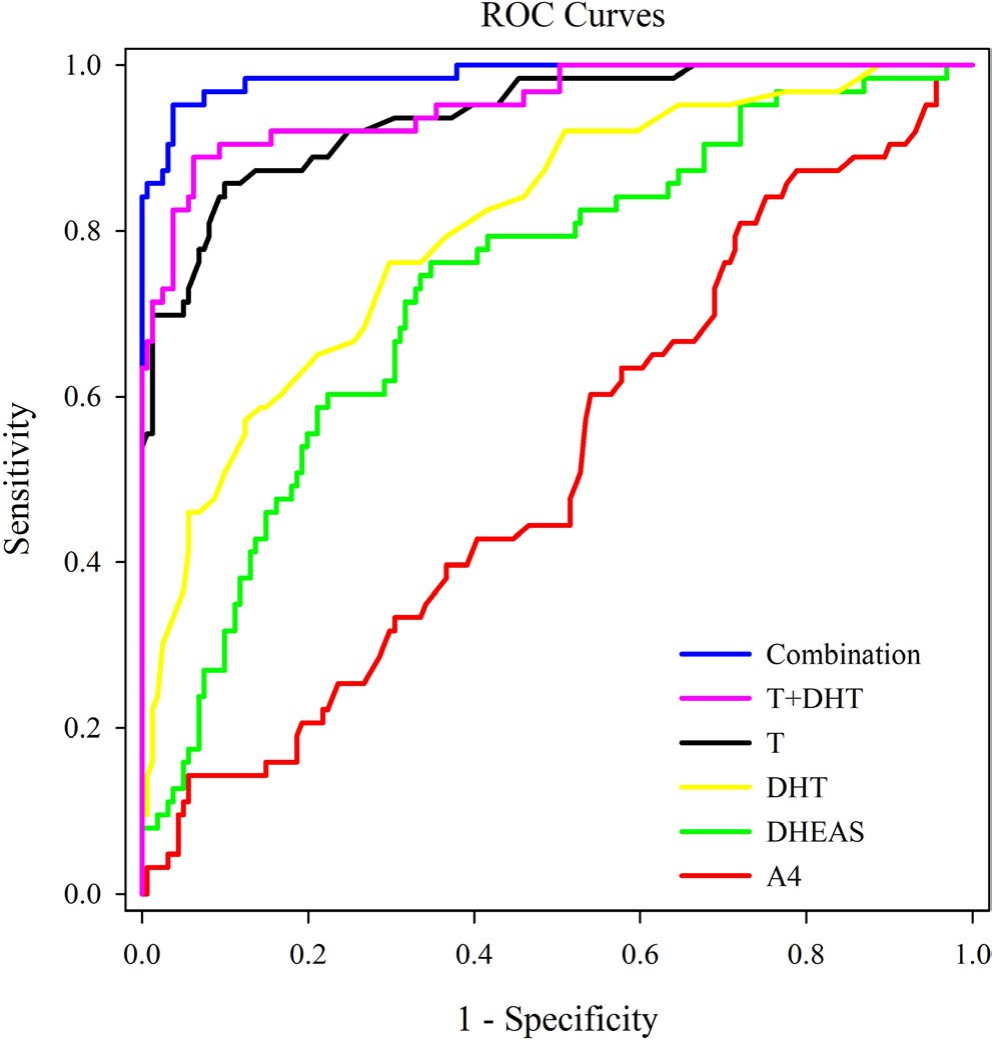
Assessment of the discriminatory performance of the four androgens using receiver operating characteristic (ROC) curves. The ROC analyses were performed with the 63 PCOS patients and 161 healthy controls. The combination detection referred to the simultaneous measurements of the four androgens of T, A4, DHEAS, and DHT

With all the PCOS (n = 63) and the healthy (n = 161) subjects enrolled, the ROC analyses showed that the AUCs of T, A4, DHEAS, and DHT were 0.94, 0.52, 0.73, and 0.81, respectively (Figure 2, Table 4). In the same ROC analysis, the AUC reached 0.99 in the case of simultaneous quantitation of T, A4, DHEAS, and DHT compared with the AUC of 0.95 for T + DHT combination, suggesting the joint detection of the four androgens was superior to any single androgen measurement (*P* < .05) or T + DHT combination in the diagnosis of PCOS (Figure 2, Table 4, and Table S1). At the same time, with the IDI evaluation of the ROC analyses, the four androgen testing panel showed better performance than any of the single andren or the T + DHT combination in discriminating PCOS patients, suggesting the clinical utility and significance of simultaneous quantifying these androgens (*P* < .05 for AUC comparison and IDI calculation in Table S1). The raw data of the four androgen measurements in both the PCOS patients and the healthy controls were available in Table S4.

As shown in Table 4, with the cutoff values determined with the highest Youden Index (sum of sensitivity and specificity minus one) in the ROC analyses, the sensitivities for T, A4, DHEAS, DHT, T + DHT, and four‐androgen combination were 0.86, 0.84, 0.76, 0.76, 0.89, and 0.95, respectively; the specificities for the above analytes or combinations were 0.90, 0.25, 0.65, 0.70, 0.94, and 0.96, respectively.

## 4 DISCUSSION

Here, we developed and validated a simultaneous quantitation LC‐MS/MS method for the four androgens and 17‐hydroxyprogesterone in female serum. With the technical superiority of LC‐MS more and more acknowledged by clinical laboratories, it has become highly recommended the sex steroids to be quantitated by LC‐MS based methods.^26^ The analysis of steroids in serum samples is especially challenging due to the low level of androgens in women. Nevertheless, it has been reported that the combination of an effective serum processing method such as optimized SPE and a sensitive LC‐MS/MS instrument increased the assay performance, allowing a low quantification limit well below that reported for the androgens (T, A4, and DHEA) in female serum.^27^, ^28^ In this study, we employed a relatively simple serum preparation method and were able to completely separate and accurately quantitate the T, A4, DHEAS, DHT, and 17‐OHP with a LC‐MS/MS procedure in 6.5 minutes. Impressively, the imprecision of this assay for all the five analytes was <9%, along with ideal LLOQ, linearity, and stability, suggesting that this was a reliable method with high suitability for wide clinical implementation.

In the same study, we also evaluated the potential clinical unitality of the four‐androgen plus 17‐OHP panel in the PCOS diagnosis. As part of the natures of PCOS, complicated, and dynamic sex hormone changes were observed in several studies with T elevation being the most prominent signature of hyperandrogenism. Free T was suggested to be even more sensitive than total T in PCOS diagnosis but its direct measurement was cumbersome and laborious.^6^, ^11^, ^18^ However, it was widely accepted that the T was by far not the only androgen increased in the PCOS patients. For instance, it was reported that almost 50% of the patients with PCOS had elevated circulating levels of DHEAS and that the combinational measurements of T and DHEAS were superior to the conventional serum T alone in hyperandrogenism evaluation.^9^, ^29^, ^30^ Furthermore, as the immediate precursor of T in its biological synthesis,^31^, ^32^ A4 accumulation was also considered as a type of androgen excess.^11^, ^33^ A prospective study showed that the diagnosis of PCOS could be increased by about 9% if A4 was included in the androgen panel.^34^ Further, it was indicated the A4 might represent a more sensitive marker for the biochemical detection of hyperandrogenemia.^11^, ^33^ DHT, formed by the 5α‐reduction of T,^17^ binds to the androgen receptor with even higher affinity when compared with T, making DHT more active within its target cells.^11^, ^17^ Therefore, DHT is one of the important targeting androgens in the diagnosis and treatment of hyperandrogenism in PCOS patients, which was also indicated that the T + DHT panel was superior to any single androgen for PCOS discrimination in present study. In addition, the 17‐OHP, another androgen analog, was the main steroid recommended to exclude the non‐classic congenital adrenal hyperplasia (NCAH) ^35^, ^36^ introduced by the 21‐hydroxylase deficiency. With significant serum accumulation of 17‐OHP, the NCAH mimics the clinical presentations of PCOS and needs to be excluded in the PCOS diagnostic diagram according the main stream relevant guidelines.^2^, ^37^, ^38^

In Guo et al's recent study, with a smaller patient cohort, also confirmed that T, A4, and DHT together might contribute to the more accurate diagnosis of PCOS.^39^ Similarly, in present study, the combinational use of the four‐androgens provided optimum PCOS discrimination with the ROC analyses with the best sensitivity and specificity, suggesting their potential diagnostic value as a testing panel, although the cutoff values for each analyte needs to be further validated in independent cohorts. In another closely related study (PCOS group, n = 152) by Handelsman et al, the authors established a relative comprehensive LC‐MS steroid panel including estradiol (E2), estrone (E1), progesterone (P4), cortisol, calculated free T, 17‐OHP, and the four androgens discussed in current study.^40^ However, according to their logistic regression study using forward stepping models, it was found that their large steroid panel was no better in diagnosing PCOS patients compared with the traditional direct testosterone immunoassay or the free androgen index, reflecting the non‐homogeneity of the presumptive PCOS clinical diagnosis with additional steroid profiling.^40^ Further evaluations with larger cohort and unified methods are needed to elucidate the apparent contradictory findings about the clinical unitality of steroids testing for PCOS discrimination.

According to the current PCOS diagnosis guideline,^38^ it is required and necessary to exclude the possibility of NCAH mimicking the symptoms of PCOS. In a recent review article comparing PCOS and NCAH,^41^ on average, 87% of NCAH patients and 25% of PCOS patients showed elevated serum 17‐OHP levels, which was the only recommended screening assay to differentiate the two distinct diseases requiring different therapeutic treatments. By contrast, the common androgens such as T and DHEAS were also elevated in NCAH, limiting their applications in differentiating NCAH from PCOS. In our study, as much as 52.4% (33/63) of the PCOS patients showed elevated 17‐OHP and had been excluded for NCAH by means such as ACTH‐stimulation, LH/FSH ratio, and other discrimination criteria.^41^ Considering the wide acceptance of measuring the 17‐OHP as a NCAH exclusion marker, it would be therefore convenient for clinical laboratories to include it in the androgen panels designed for PCOS diagnosis.

## 5 CONCLUSION

In summary, a laboratory‐developed assay for simultaneous quantitation of the four androgens and 17‐OHP associated with PCOS was validated with good performance. More importantly, not only did this testing panel provided better diagnostic power for PCOS with the combinational measurements of T, A4, DHEAS, and DHT, it also added the convenience of simultaneous screening for NCAH which should be excluded in PCOS diagnosis.

## CONFLICT OF INTEREST

The authors state that there are no conflicts of interest with regard to publication of this article.

## Supporting information

Figure S1‐S2

Table S1‐S4

## References

[1] Norman RJ , Dewailly D , Legro RS , Hickey TE . Polycystic ovary syndrome. The Lancet. 2007;370(9588):685‐697. 10.1016/S0140-6736(07)61345-2 17720020

[2] Teede HJ , Misso ML , Costello MF , et al. Recommendations from the international evidence‐based guideline for the assessment and management of polycystic ovary syndrome. Hum Reprod. 2018;33(9):1602‐1618. 30052961 10.1093/humrep/dey256 PMC6112576

[3] Escobar‐Morreale HF . Polycystic ovary syndrome: definition, aetiology, diagnosis and treatment. Nat Rev Endocrinol. 2018;14(5):270‐284. 29569621 10.1038/nrendo.2018.24

[4] Ehrmann DA . Polycystic ovary syndrome. N Engl J Med. 2005;352(12):1223‐1236. 15788499 10.1056/NEJMra041536

[5] Rotterdam EA‐SPCWG . Revised 2003 consensus on diagnostic criteria and long‐term health risks related to polycystic ovary syndrome. Fertil Steril. 2004;81(1):19‐25. 10.1016/j.fertnstert.2003.10.004 14711538

[6] Goodman NF , Cobin RH , Futterweit W , et al. American Association of Clinical Endocrinologists, American College of Endocrinology, and Androgen Excess and PCOS Society Disease State Clinical Review: Guide to the best practices in the evaluation and treatment of polycystic ovary syndrome‐part 1. Endocr Pract. 2015;21(11):1291‐1300. 26509855 10.4158/EP15748.DSC

[7] Rosenfield RL , Ehrmann DA . The pathogenesis of polycystic ovary syndrome (PCOS): the hypothesis of PCOS as functional ovarian hyperandrogenism revisited. Endocr Rev. 2016;37(5):467‐520. 27459230 10.1210/er.2015-1104 PMC5045492

[8] Goodman NF , Cobin RH , Futterweit W , et al. American Association of Clinical Endocrinologists, American College of Endocrinology, and Androgen Excess and Pcos Society Disease State Clinical Review: guide to the best practices in the evaluation and treatment of polycystic ovary syndrome‐part 2. Endocr Pract. 2015;21(12):1415‐1426. 26642102 10.4158/EP15748.DSCPT2

[9] Carmina E . Ovarian and adrenal hyperandrogenism. Ann N Y Acad Sci. 2006;1092:130‐137. 17308139 10.1196/annals.1365.011

[10] Yildiz BO , Bozdag G , Yapici Z , Esinler I , Yarali H . Prevalence, phenotype and cardiometabolic risk of polycystic ovary syndrome under different diagnostic criteria. Hum Reprod. 2012;27(10):3067‐3073. 22777527 10.1093/humrep/des232

[11] Azziz R , Carmina E , Dewailly D , et al. The Androgen Excess and PCOS Society criteria for the polycystic ovary syndrome: the complete task force report. Fertil Steril. 2009;91(2):456‐488. 18950759 10.1016/j.fertnstert.2008.06.035

[12] Rosner W , Auchus RJ , Azziz R , Sluss PM , Raff H . Position statement: utility, limitations, and pitfalls in measuring testosterone: an Endocrine Society position statement. J Clin Endocrinol Metab. 2007;92(2):405‐413. 17090633 10.1210/jc.2006-1864

[13] Stanczyk FZ , Jurow J , Hsing AW . Limitations of direct immunoassays for measuring circulating estradiol levels in postmenopausal women and men in epidemiologic studies. Cancer Epidemiol Biomarkers Prev. 2010;19(4):903‐906. 20332268 10.1158/1055-9965.EPI-10-0081

[14] Soldin SJ , Soldin OP . Steroid hormone analysis by tandem mass spectrometry. Clin Chem. 2009;55(6):1061‐1066. 19325015 10.1373/clinchem.2007.100008 PMC3634331

[15] McDonald JG , Matthew S , Auchus RJ . Steroid profiling by gas chromatography‐mass spectrometry and high performance liquid chromatography‐mass spectrometry for adrenal diseases. Horm Cancer. 2011;2(6):324‐332. 22170384 10.1007/s12672-011-0099-x PMC3437926

[16] Gallagher LMOL , Keevil BG . Simultaneous determination of androstenedione and testosterone in human serum by liquid chromatography‐tandem mass spectrometry. Ann Clin Biochem. 2007;44(1):48‐56. 17270092 10.1258/000456307779595922

[17] Munzker J , Hofer D , Trummer C , et al. Testosterone to dihydrotestosterone ratio as a new biomarker for an adverse metabolic phenotype in the polycystic ovary syndrome. J Clin Endocrinol Metab. 2015;100(2):653‐660. 25387259 10.1210/jc.2014-2523

[18] Bui HN , Sluss PM , Hayes FJ , et al. Testosterone, free testosterone, and free androgen index in women: reference intervals, biological variation, and diagnostic value in polycystic ovary syndrome. Clin Chim Acta. 2015;450:227‐232. 26327459 10.1016/j.cca.2015.08.019

[19] Keevil B . Steroid mass spectrometry for the diagnosis of PCOS. Med Sci (Basel). 2019;7(7):78. 10.3390/medsci7070078 PMC6681326 31295971

[20] Kannenberg F , Fobker M , Schulte E , et al. The Simultaneous measurement of serum testosterone and 5alpha‐dihydrotestosterone by gas chromatography‐mass spectrometry (GC‐MS). Clin Chim Acta. 2018;476:15‐24. 29122541 10.1016/j.cca.2017.10.030

[21] Shiraishi S , Lee PW , Leung A , Goh VH , Swerdloff RS , Wang C . Simultaneous measurement of serum testosterone and dihydrotestosterone by liquid chromatography‐tandem mass spectrometry. Clin Chem. 2008;54(11):1855‐1863. 18801940 10.1373/clinchem.2008.103846

[22] CLSI . Liquid‐Chromatography‐Mass Spectrometry Methods; Approved Guideline. CLSI Document C62‐A. Wayne, PA: Clinical and Laboratory Standards Institute; 2014.

[23] Matuszewski BK , Constanzer ML , Chavez‐Eng CM . Strategies for the assessment of matrix effect in quantitative bioanalytical methods based on HPLC‐MS/MS. Anal Chem. 2003;75(13):3019‐3030. 12964746 10.1021/ac020361s

[24] Clinical and Laboratory Standard Institute . Defining, Establishing, and Verifying Reference Intervals in the Clinical Laboratory; Approved Guideline. 3rd ed Wayne, PA: Clinical and Laboratory Standard Institute; 2008.

[25] Pencina MJ , D'Agostino RB Sr , D'Agostino RB Jr , Vasan RS . Evaluating the added predictive ability of a new marker: from area under the ROC curve to reclassification and beyond. Stat Med. 2008;27(2):157‐172. 17569110 10.1002/sim.2929

[26] Handelsman DJ , Wartofsky L . Requirement for mass spectrometry sex steroid assays in the Journal of Clinical Endocrinology and Metabolism. J Clin Endocrinol Metab. 2013;98(10):3971‐3973. 24098015 10.1210/jc.2013-3375

[27] Xu W , Li H , Guan Q , Shen Y , Cheng L . A rapid and simple liquid chromatography‐tandem mass spectrometry method for the measurement of testosterone, androstenedione, and dehydroepiandrosterone in human serum. J Clin Lab Anal. 2017;31(5):e22102. 10.1002/jcla.22102 PMC6817279 27911021

[28] Buttler RM , Martens F , Kushnir MM , Ackermans MT , Blankenstein MA , Heijboer AC . Simultaneous measurement of testosterone, androstenedione and dehydroepiandrosterone (DHEA) in serum and plasma using Isotope‐Dilution 2‐Dimension Ultra High Performance Liquid‐Chromatography Tandem Mass Spectrometry (ID‐LC‐MS/MS). Clin Chim Acta. 2015;438:157‐159. 25172041 10.1016/j.cca.2014.08.023

[29] Guducu N , Kutay SS , Gormus U , Kavak ZN , Dunder I . High DHEAS/free testosterone ratio is related to better metabolic parameters in women with PCOS. Gynecol Endocrinol. 2015;31(6):495‐500. 25986306 10.3109/09513590.2015.1022862

[30] Goodarzi MO , Carmina E , Azziz R . DHEA, DHEAS and PCOS. J Steroid Biochem Mol Biol. 2015;145:213‐225. 25008465 10.1016/j.jsbmb.2014.06.003

[31] Huerta R , Dewailly D , Decanter C , Knochenhauer ES , Boots LR , Azziz R . 11beta‐hydroxyandrostenedione and delta5‐androstenediol as markers of adrenal androgen production in patients with 21‐hydroxylase‐deficient nonclassic adrenal hyperplasia. Fertil Steril. 1999;72(6):996‐1000. 10593370 10.1016/s0015-0282(99)00402-1

[32] Franik G , Maksym M , Owczarek AJ , Chudek J , Madej P , Olszanecka‐Glinianowicz M . Estradiol/testosterone and estradiol/androstenedione indexes and nutritional status in PCOS women – a pilot study. Eur J Obstet Gynecol Reprod Biol. 2019;242:166‐169. 31600717 10.1016/j.ejogrb.2019.05.045

[33] O'Reilly MW , Taylor AE , Crabtree NJ , et al. Hyperandrogenemia predicts metabolic phenotype in polycystic ovary syndrome: the utility of serum androstenedione. J Clin Endocrinol Metab. 2014;99(3):1027‐1036. 24423344 10.1210/jc.2013-3399 PMC3955250

[34] Knochenhauer ESKT , Kahsar‐Miller M , Waggoner W , Boots LR , Azziz R . Prevalence of the polycystic ovary syndrome in unselected black and white women of the southeastern United States: a prospective study. J Clin Endocrinol Metab. 1998;83(9):3078‐3082. 9745406 10.1210/jcem.83.9.5090

[35] Carmina E . Hirsutism: investigation and management. Expert Rev Endocrinol Metab. 2010;5(2):189‐195. 30764045 10.1586/eem.09.73

[36] Maas KH , Chuan SS , Cook‐Andersen H , Su HI , Duleba A , Chang RJ . Relationship between 17‐hydroxyprogesterone responses to human chorionic gonadotropin and markers of ovarian follicle morphology in women with polycystic ovary syndrome. J Clin Endocrinol Metab. 2015;100(1):293‐300. 25313914 10.1210/jc.2014-2956 PMC4283019

[37] Wang FF , Pan JX , Wu Y , Zhu YH , Hardiman PJ , Qu F . American, European, and Chinese practice guidelines or consensuses of polycystic ovary syndrome: a comparative analysis. J Zhejiang Univ Sci B. 2018;19(5):354‐363. 29732746 10.1631/jzus.B1700074 PMC5962512

[38] Legro RS , Arslanian SA , Ehrmann DA , et al. Diagnosis and treatment of polycystic ovary syndrome: an Endocrine Society clinical practice guideline. J Clin Endocrinol Metab. 2013;98(12):4565‐4592. 24151290 10.1210/jc.2013-2350 PMC5399492

[39] Wang Z , Wang H , Peng Y , et al. A liquid chromatography‐tandem mass spectrometry (LC‐MS/MS)‐based assay to profile 20 plasma steroids in endocrine disorders. Clin Chem Lab Med (CCLM). 2020 https://pubmed.ncbi.nlm.nih.gov/32084000/. [Epub ahead of print]. 10.1515/cclm-2019-0869 32084000

[40] Handelsman DJ , Teede HJ , Desai R , Norman RJ , Moran LJ . Performance of mass spectrometry steroid profiling for diagnosis of polycystic ovary syndrome. Hum Reprod. 2017;32(2):418‐422. 27999117 10.1093/humrep/dew328

[41] Papadakis G , Kandaraki EA , Tseniklidi E , Papalou O , Diamanti‐Kandarakis E . Polycystic ovary syndrome and NC‐CAH: distinct characteristics and common findings. A systematic review. Front Endocrinol (Lausanne). 2019;10:388. 31275245 10.3389/fendo.2019.00388 PMC6593353

