# Supplementary material for: Simultaneous quantitation of four androgens and 17‐hydroxyprogesterone in polycystic ovarian syndrome patients by LC‐MS/MS
Source: J Clin Lab Anal. 2020 Aug 21;34(12):e23539. doi: 10.1002/jcla.23539 (PMC7755789; doi:10.1002/jcla.23539)
Supplement: Supplementary file 1 — Figure S1‐S2 [file JCLA-34-e23539-s001.docx]

**Supplementary Figure legends**

**Supplementary Figure S1** The chromatogram with the present LC-MS/MS method using a double blank sample that was prepared with hormone free human plasma with no reference or internal standards added.


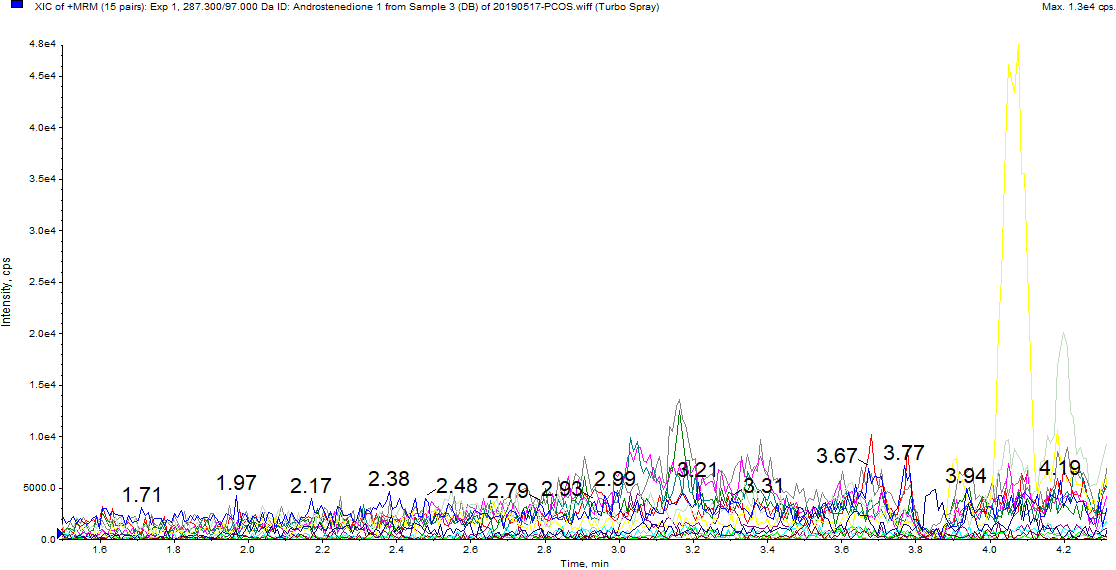


**Supplementary Figure S2**

The extracted MS/MS spectra for testosterone (A), androstenedione (B), dehydroepiandrosterone sulfate (C), dihydrotestosterone (D), and 17-hydroxyprogesterone (E).


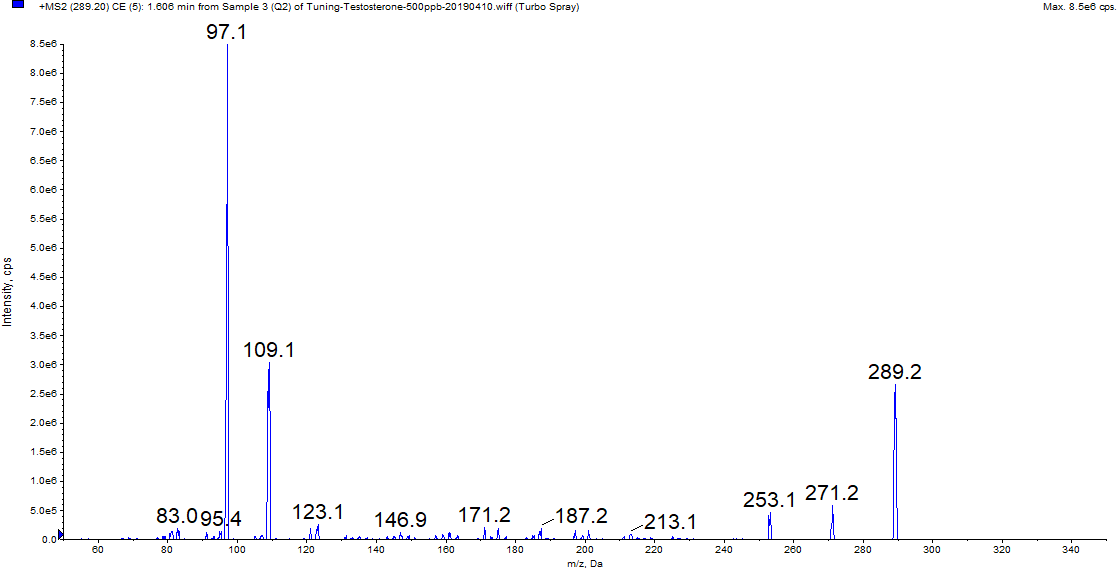


A: MS/MS spectra for testosterone


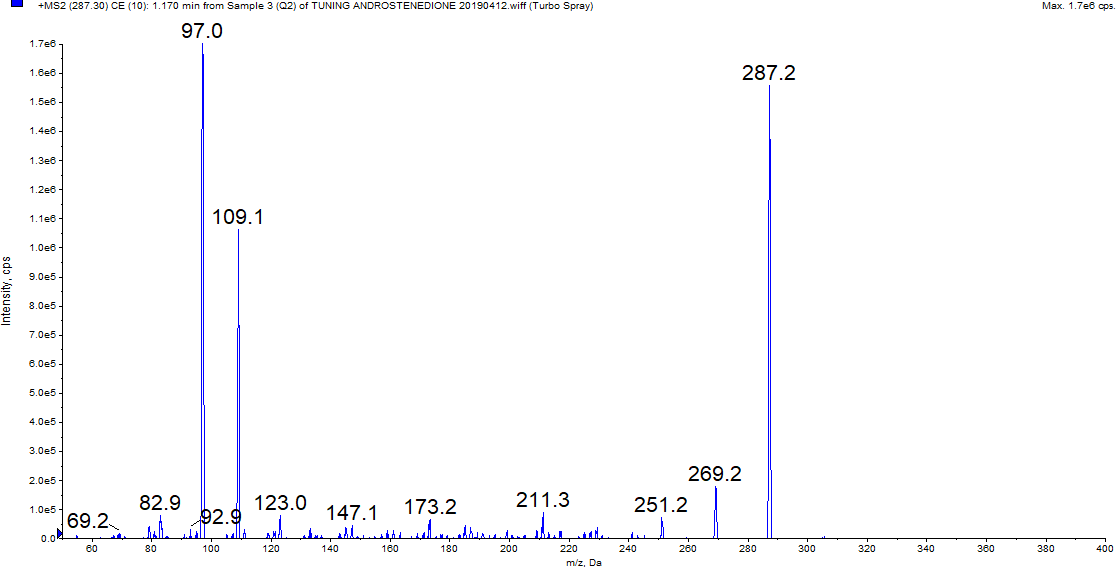


B: MS/MS spectra for androstenedione


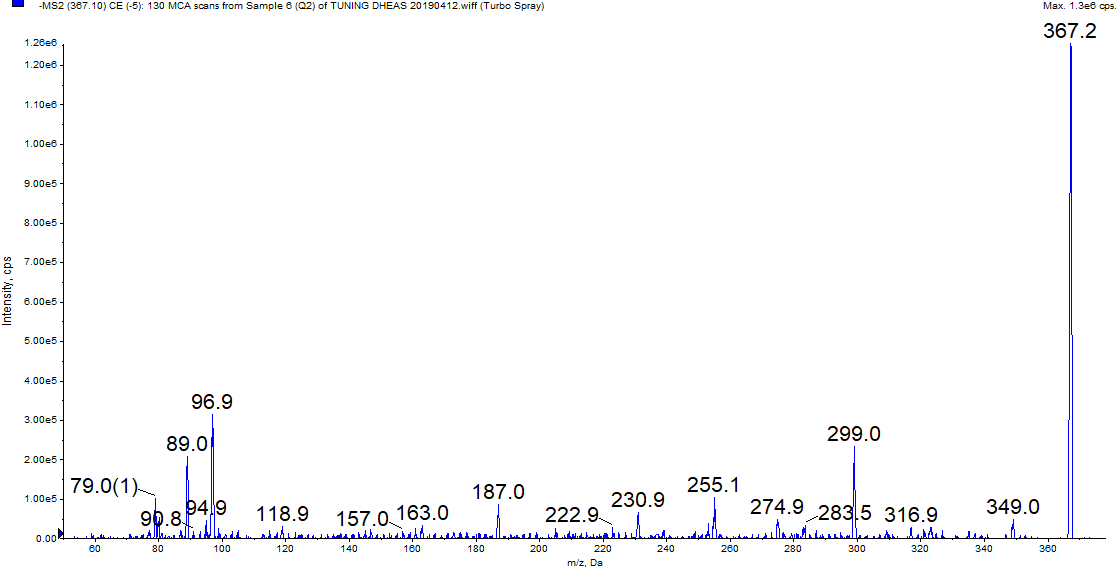


C: MS/MS spectra for dehydroepiandrosterone sulfate


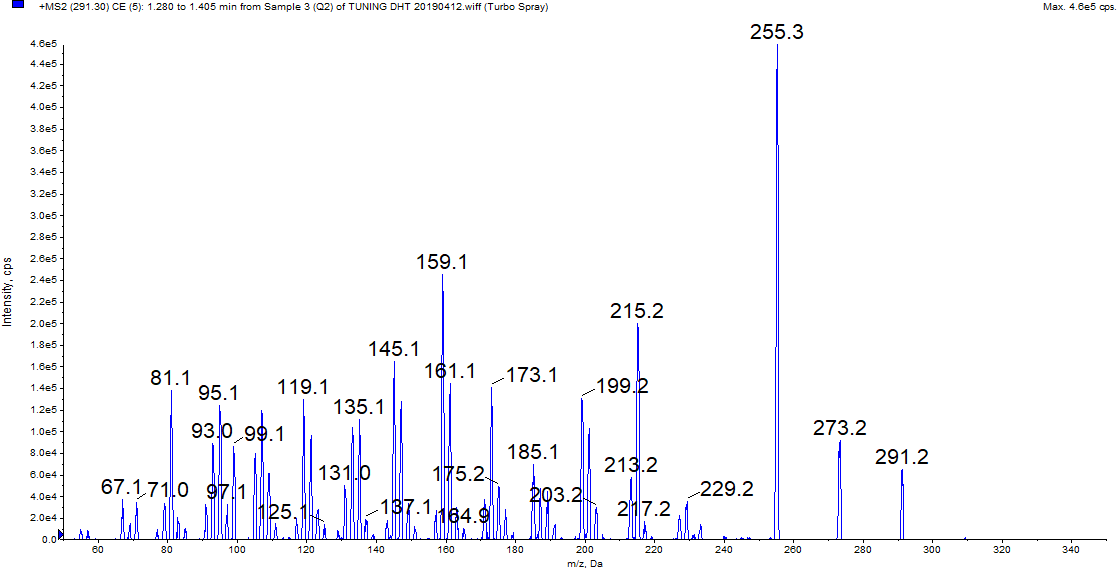


D: MS/MS spectra for dihydrotestosterone


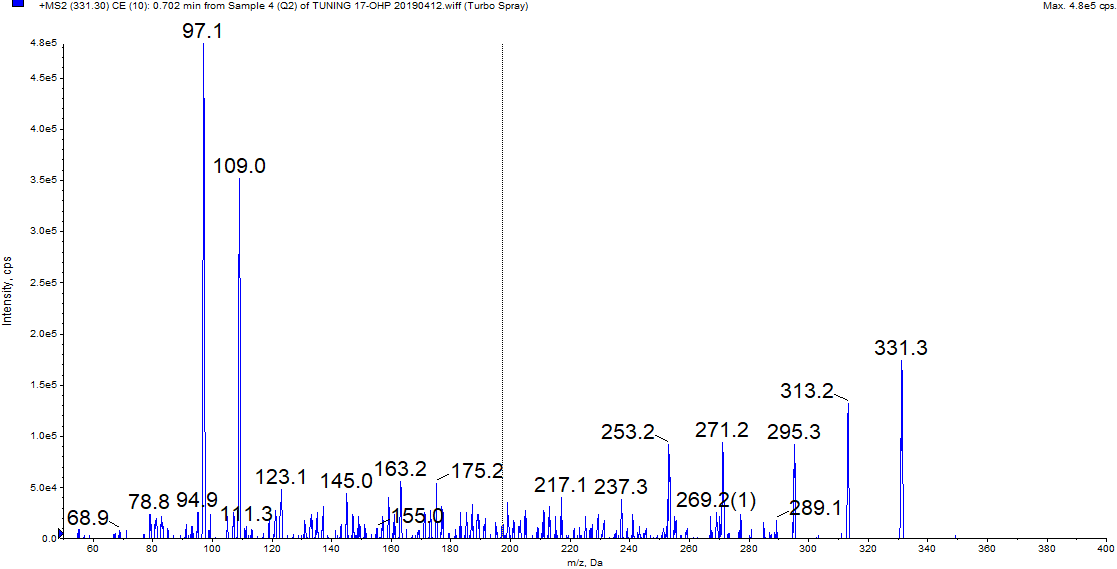


E: MS/MS spectra for 17-hydroxyprogesterone
