# Supplementary material for: Simultaneous quantitation of four androgens and 17‐hydroxyprogesterone in polycystic ovarian syndrome patients by LC‐MS/MS
Source: J Clin Lab Anal. 2020 Aug 21;34(12):e23539. doi: 10.1002/jcla.23539 (PMC7755789; doi:10.1002/jcla.23539)
Supplement: Supplementary file 2 — Table S1‐S4 [file JCLA-34-e23539-s002.docx]

**Supplementary Table S1** The AUC comparison in the ROC analyses and the IDI evaluation in the four-androgen PCOS diagnosis

|  |  |  |  |  |  |  |
| --- | --- | --- | --- | --- | --- | --- |
| Comparison Pairs | Area Difference | Standard Error | 95% Confidence Interval | p value^a^ | IDI^b^ | p value^c^ |
| T, A4 | 0.42 | 0.04 | 0.34 to 0.49 | < 0.0001 | ND^d^ |  |
| T, DHEAS | 0.21 | 0.04 | 0.14 to 0.28 | < 0.0001 | ND |  |
| T, DHT | 0.13 | 0.04 | 0.06 to 0.20 | 0.0002 | ND |  |
| T, Combination | -0.05 | 0.02 | -0.08 to -0.02 | 0.0037 | 0.49 | <0.001 |
| T, T+DHT | -0.01 | 0.01 | -0.03 to 0.00 | 0.1288 | 0.34 | <0.001 |
| A4, DHEAS | -0.21 | 0.04 | -0.29 to -0.13 | < 0.0001 | ND |  |
| A4, DHT | -0.29 | 0.05 | -0.38 to -0.19 | < 0.0001 | ND |  |
| A4, Combination | -0.47 | 0.04 | -0.55 to -0.38 | < 0.0001 | 0.82 | <0.001 |
| A4, T+DHT | -0.43 | 0.04 | -0.51 to -0.36 | < 0.0001 | 0.68 | <0.001 |
| DHEAS, DHT | -0.07 | 0.04 | -0.16 to 0.01 | 0.0997 | ND |  |
| DHEAS, Combination | -0.26 | 0.04 | -0.33 to -0.18 | < 0.0001 | 0.72 | <0.001 |
| DHEAS, T+DHT | -0.22 | 0.04 | -0.29 to -0.15 | < 0.0001 | 0.57 | <0.001 |
| DHT, Combination | -0.18 | 0.03 | -0.25 to -0.12 | < 0.0001 | 0.65 | <0.001 |
| DHT, T+DHT | -0.15 | 0.03 | -0.21 to -0.09 | < 0.0001 | 0.51 | <0.001 |
| Combination, T+DHT | 0.03 | 0.02 | 0.00 to 0.06 | 0.0264 | 0.15 | <0.001 |
| a: p value calculated in AUC (area under curve) of the ROC analyses; b IDI, integrated discrimination improvement; c: p value calculated in the IDI evaluation; d: ND, not determined. | | | | | | |

**Supplementary Table S2** The 95% confidence intervals for the calibrators in the linearity validation

|  |  |  |  |  |  |  |  |  |  |  |  |  |  |  |
| --- | --- | --- | --- | --- | --- | --- | --- | --- | --- | --- | --- | --- | --- | --- |
| Standard (STD) | T (ng/ml) | |  | A4 (ng/ml) | |  | DHEAS (ng/ml) | |  | DHT (ng/ml) | |  | 17-OHP (ng/ml) | |
|  | NC^a^ | 95% CI^b^ |  | NC | 95% CI |  | NC | 95% CI |  | NC | 95% CI |  | NC | 95% CI |
| STD1 | 0.05 | 0.04-0.06 |  | 0.1 | 0.09-0.11 |  | 10 | 9.3-10.8 |  | 0.05 | 0.04-0.06 |  | 0.05 | 0.05-0.05 |
| STD2 | 0.1 | 0.10-0.10 |  | 0.2 | 0.20-0.22 |  | 20 | 17.3-21.7 |  | 0.1 | 0.10-0.11 |  | 0.1 | 0.09-0.12 |
| STD3 | 0.2 | 0.19-0.22 |  | 0.4 | 0.39-0.41 |  | 40 | 37.2-44.4 |  | 0.2 | 0.17-0.22 |  | 0.2 | 0.19-0.23 |
| STD4 | 0.6 | 0.58-0.68 |  | 1.2 | 1.20-1.28 |  | 120 | 115.0-133.0 |  | 0.6 | 0.61-0.64 |  | 0.6 | 0.59-0.70 |
| STD5 | 3 | 2.78-3.17 |  | 6 | 5.73-6.47 |  | 600 | 561.2-657.5 |  | 3 | 2.89-3.14 |  | 3 | 2.76-3.01 |
| STD6 | 6 | 5.11-6.19 |  | 12 | 9.49-13.13 |  | 1200 | 957.4-1324.5 |  | 6 | 5.10-6.16 |  | 6 | 4.71-6.17 |
| a: NC, nominal concentration; b: CI, confidence interval. | | | | | |  |  |  |  |  |  |  |  |  |

**Supplementary Table S3** The 95% confidence intervals for QC-L, QC-M and QC-H in the precision and sensitivity validations

|  |  |  |  |  |  |  |  |  |  |  |
| --- | --- | --- | --- | --- | --- | --- | --- | --- | --- | --- |
| Analytes | QC-L, ng/ml | |  | QC-M, ng/ml | |  | QC-H, ng/ml | |  | LLOQ^a^, ng/ml |
|  | Intra-assay | Inter-assay |  | Intra-assay | Inter-assay |  | Intra-assay | Inter-assay |  |  |
| T | 0.09-0.12 | 0.08-0.11 |  | 0.57-0.69 | 0.55-0.68 |  | 4.31-5.06 | 4.16-5.21 |  | 0.04-0.06 |
| A4 | 0.16-0.23 | 0.17-0.23 |  | 1.14-1.34 | 1.15-1.36 |  | 8.61-10.38 | 8.52-10.47 |  | 0.05-0.05 |
| DHEAS | 17.9-21.9 | 18.5-23.6 |  | 120.8-142.0 | 112.3-140.5 |  | 909.8-1112.9 | 899.2-1123.6 |  | 3.6-6.5 |
| DHT | 0.08-0.12 | 0.08-0.12 |  | 0.57-0.66 | 0.55-0.70 |  | 4.20-5.37 | 4.27-5.30 |  | 0.02-0.05 |
| 17-OHP | 0.09-0.11 | 0.09-0.12 |  | 0.61-0.70 | 0.56-0.70 |  | 4.55-5.26 | 4.51-5.30 |  | 0.02-0.05 |
| a: LLOQ, lower limit of quantitation. | | |  |  |  |  |  |  |  |  |
|  |  |  |  |  |  |  |  |  |  |  |

**Supplementary Table S4** The raw data used in ROC analyses for PCOS diagnosis

|  |  |  |  |  |
| --- | --- | --- | --- | --- |
| Group | T (ng/ml) | A4 (ng/ml) | DHEAS (ng/ml) | DHT (ng/ml) |
| PCOS | 0.68 | 2.07 | 1613.2 | 0.37 |
| PCOS | 0.45 | 0.34 | 519.3 | 0.41 |
| PCOS | 1.32 | 0.83 | 1531.6 | 0.1 |
| PCOS | 1.33 | 1.3 | 2061.6 | 0.7 |
| PCOS | 1.82 | 2.24 | 1657.0 | 0.75 |
| PCOS | 1.68 | 2.88 | 1422.6 | 0.5 |
| PCOS | 2.37 | 2.21 | 2918.1 | 0.72 |
| PCOS | 2.29 | 1.21 | 1133.9 | 0.47 |
| PCOS | 1.69 | 1.34 | 1840.4 | 0.08 |
| PCOS | 1.08 | 1.58 | 2998.2 | 0.27 |
| PCOS | 1.72 | 1.25 | 1567.0 | 0.68 |
| PCOS | 0.35 | 0.39 | 678.3 | 0.15 |
| PCOS | 1.35 | 2.32 | 1161.5 | 0.26 |
| PCOS | 1.44 | 1.05 | 1813.0 | 0.35 |
| PCOS | 0.69 | 0.82 | 1622.8 | 0.94 |
| PCOS | 1.15 | 1.15 | 1480.2 | 0.23 |
| PCOS | 1.1 | 1.47 | 2039.8 | 0.54 |
| PCOS | 1 | 0.83 | 677.1 | 0.41 |
| PCOS | 0.95 | 0.93 | 1326.2 | 0.16 |
| PCOS | 1.57 | 2.12 | 1191.7 | 0.12 |
| PCOS | 1.25 | 1.62 | 1645.6 | 0.32 |
| PCOS | 0.99 | 2.09 | 1987.0 | 0.22 |
| PCOS | 0.37 | 0.82 | 1730.5 | 0.15 |
| PCOS | 0.8 | 0.98 | 876.9 | 0.17 |
| PCOS | 1.3 | 1.49 | 1401.0 | 0.5 |
| PCOS | 1.26 | 0.72 | 725.4 | 0.19 |
| PCOS | 0.48 | 1.04 | 1026.0 | 0.25 |
| PCOS | 1.01 | 1.77 | 4154.0 | 0.57 |
| PCOS | 1.47 | 1.45 | 627.1 | 0.19 |
| PCOS | 0.65 | 1.39 | 974.2 | 0.36 |
| PCOS | 1.93 | 1.22 | 1735.2 | 0.31 |
| PCOS | 1.37 | 0.76 | 780.5 | 0.12 |
| PCOS | 1.52 | 2.23 | 1847.3 | 0.37 |
| PCOS | 1.43 | 1.35 | 1382.8 | 0.36 |
| PCOS | 0.99 | 0.99 | 2404.7 | 0.38 |
| PCOS | 1.99 | 1.04 | 2695.0 | 0.62 |
| PCOS | 1.37 | 1.61 | 1156.6 | 0.29 |
| PCOS | 0.71 | 0.85 | 1272.3 | 0.32 |
| PCOS | 1.34 | 1.06 | 2188.2 | 0.3 |
| PCOS | 0.43 | 0.62 | 728.8 | 0.36 |
| PCOS | 0.54 | 1.06 | 1461.5 | 0.08 |
| PCOS | 1.38 | 0.84 | 960.3 | 0.24 |
| PCOS | 0.78 | 1.15 | 1745.9 | 0.26 |
| PCOS | 2 | 0.79 | 1355.0 | 0.59 |
| PCOS | 0.25 | 0.76 | 1142.2 | 0.31 |
| PCOS | 0.83 | 1.39 | 2569.4 | 0.57 |
| PCOS | 1.01 | 1.24 | 1357.5 | 0.37 |
| PCOS | 0.75 | 1.02 | 1134.2 | 0.44 |
| PCOS | 1.53 | 0.9 | 1171.5 | 0.33 |
| PCOS | 1.92 | 1.09 | 1099.8 | 0.22 |
| PCOS | 1.44 | 1.05 | 1813.0 | 0.35 |
| PCOS | 0.79 | 1.04 | 1284.9 | 0.16 |
| PCOS | 0.94 | 1.04 | 1107.4 | 0.22 |
| PCOS | 1.03 | 0.8 | 1506.0 | 0.41 |
| PCOS | 0.9 | 0.48 | 306.8 | 0.18 |
| PCOS | 0.45 | 0.78 | 1829.8 | 0.41 |
| PCOS | 0.35 | 0.39 | 678.3 | 0.15 |
| PCOS | 0.98 | 0.71 | 1294.4 | 0.26 |
| PCOS | 1.56 | 1.02 | 3219.8 | 0.35 |
| PCOS | 2.33 | 2.96 | 1862.1 | 0.23 |
| PCOS | 1.08 | 0.5 | 1079.0 | 0.18 |
| PCOS | 1.61 | 0.58 | 1034.7 | 0.31 |
| PCOS | 0.62 | 0.48 | 784.8 | 0.56 |
| Healthy | 0.27 | 0.85 | 992.7 | 0.16 |
| Healthy | 0.52 | 1.05 | 1108.5 | 0.18 |
| Healthy | 0.09 | 0.41 | 567.5 | 0.09 |
| Healthy | 0.28 | 0.99 | 1506.4 | 0.25 |
| Healthy | 0.22 | 0.73 | 1401.9 | 0.11 |
| Healthy | 0.45 | 1.5 | 1012.2 | 0.31 |
| Healthy | 0.48 | 2.06 | 1038.5 | 0.18 |
| Healthy | 1.02 | 2.04 | 1351.6 | 0.28 |
| Healthy | 0.51 | 1.89 | 998.0 | 0.19 |
| Healthy | 0.2 | 1.07 | 711.1 | 0.13 |
| Healthy | 0.23 | 0.81 | 1074.4 | 0.17 |
| Healthy | 0.34 | 1.08 | 975.5 | 0.08 |
| Healthy | 0.48 | 0.96 | 625.7 | 0.14 |
| Healthy | 0.54 | 1.97 | 1063.2 | 0.26 |
| Healthy | 0.15 | 0.71 | 502.3 | 0.18 |
| Healthy | 0.33 | 1.48 | 1191.1 | 0.17 |
| Healthy | 0.16 | 0.62 | 919.4 | 0.22 |
| Healthy | 0.28 | 0.96 | 1002.5 | 0.11 |
| Healthy | 0.23 | 1.09 | 1877.6 | 0.41 |
| Healthy | 0.42 | 1.67 | 1027.2 | 0.11 |
| Healthy | 0.24 | 0.61 | 996.4 | 0.21 |
| Healthy | 0.15 | 0.78 | 1661.6 | 0.14 |
| Healthy | 0.42 | 1.32 | 335.6 | 0.25 |
| Healthy | 0.24 | 0.89 | 821.4 | 0.06 |
| Healthy | 0.18 | 0.76 | 725.1 | 0.08 |
| Healthy | 0.35 | 1.27 | 2157.3 | 0.11 |
| Healthy | 0.18 | 0.76 | 2112.2 | 0.06 |
| Healthy | 0.85 | 2.33 | 2027.3 | 0.31 |
| Healthy | 0.5 | 2.1 | 1306.3 | 0.15 |
| Healthy | 0.19 | 0.84 | 1364.7 | 0.08 |
| Healthy | 0.31 | 0.94 | 1575.1 | 0.02 |
| Healthy | 0.24 | 0.89 | 550.4 | 0.2 |
| Healthy | 0.55 | 1.4 | 1817.6 | 0.12 |
| Healthy | 0.38 | 1.28 | 1025.1 | 0.09 |
| Healthy | 0.2 | 0.62 | 430.2 | 0.11 |
| Healthy | 0.39 | 2.26 | 985.0 | 0.1 |
| Healthy | 0.21 | 0.58 | 781.3 | 0.17 |
| Healthy | 0.36 | 1.26 | 267.4 | 0.22 |
| Healthy | 0.43 | 1.75 | 1042.1 | 0.13 |
| Healthy | 0.16 | 0.66 | 590.9 | 0.18 |
| Healthy | 0.35 | 1.09 | 1241.8 | 0.09 |
| Healthy | 0.25 | 1.01 | 1204.9 | 0.09 |
| Healthy | 0.63 | 1.92 | 1136.6 | 0.25 |
| Healthy | 0.54 | 0.61 | 440.8 | 0.17 |
| Healthy | 0.45 | 1.34 | 1634.7 | 0.07 |
| Healthy | 0.14 | 0.32 | 244.0 | 0.2 |
| Healthy | 0.5 | 1.81 | 990.1 | 0.09 |
| Healthy | 0.09 | 0.27 | 125.7 | 0.26 |
| Healthy | 0.39 | 1.01 | 1103.5 | 0.12 |
| Healthy | 0.44 | 1.08 | 640.6 | 0.25 |
| Healthy | 0.79 | 1.83 | 1155.3 | 0.11 |
| Healthy | 0.39 | 1.08 | 615.2 | 0.25 |
| Healthy | 0.3 | 0.73 | 555.7 | 0.07 |
| Healthy | 0.32 | 0.72 | 934.4 | 0.14 |
| Healthy | 0.39 | 1.39 | 1299.4 | 0.17 |
| Healthy | 0.15 | 0.65 | 426.6 | 0.11 |
| Healthy | 0.43 | 1.11 | 636.3 | 0.31 |
| Healthy | 0.71 | 1.25 | 786.2 | 0.1 |
| Healthy | 0.07 | 0.38 | 545.1 | 0.12 |
| Healthy | 0.86 | 1.24 | 441.5 | 0.14 |
| Healthy | 0.35 | 0.93 | 592.7 | 0.08 |
| Healthy | 0.37 | 0.88 | 1026.0 | 0.07 |
| Healthy | 0.17 | 0.64 | 434.6 | 0.22 |
| Healthy | 0.49 | 2.61 | 1755.5 | 0.09 |
| Healthy | 0.3 | 0.96 | 525.3 | 0.37 |
| Healthy | 0.74 | 2.04 | 1962.6 | 0.11 |
| Healthy | 0.14 | 0.61 | 867.8 | 0.07 |
| Healthy | 0.22 | 0.93 | 1771.5 | 0.27 |
| Healthy | 0.53 | 1.19 | 351.1 | 0.06 |
| Healthy | 0.15 | 0.69 | 581.8 | 0.16 |
| Healthy | 0.17 | 0.5 | 502.6 | 0.19 |
| Healthy | 0.41 | 1.14 | 1020.1 | 0.25 |
| Healthy | 0.26 | 0.8 | 663.9 | 0.07 |
| Healthy | 0.32 | 1.14 | 856.6 | 0.14 |
| Healthy | 0.38 | 1 | 1192.9 | 0.1 |
| Healthy | 0.4 | 1.15 | 1390.1 | 0.07 |
| Healthy | 1.03 | 2.33 | 1798.3 | 0.17 |
| Healthy | 0.23 | 1.2 | 1230.4 | 0.12 |
| Healthy | 0.19 | 0.64 | 415.3 | 0.2 |
| Healthy | 0.36 | 1.17 | 1503.7 | 0.08 |
| Healthy | 0.37 | 1.35 | 408.2 | 0.1 |
| Healthy | 0.17 | 0.59 | 952.2 | 0.29 |
| Healthy | 0.28 | 1.24 | 1223.5 | 0.1 |
| Healthy | 0.27 | 0.84 | 850.3 | 0.13 |
| Healthy | 0.22 | 0.77 | 1018.3 | 0.33 |
| Healthy | 0.23 | 0.9 | 759.5 | 0.31 |
| Healthy | 0.33 | 1.22 | 1277.6 | 0.18 |
| Healthy | 0.24 | 1.07 | 755.8 | 0.05 |
| Healthy | 0.27 | 1.58 | 876.5 | 0.06 |
| Healthy | 0.32 | 1.39 | 2444.2 | 0.32 |
| Healthy | 0.37 | 1.15 | 998.3 | 0.1 |
| Healthy | 0.46 | 1.45 | 1458.0 | 0.2 |
| Healthy | 0.32 | 0.99 | 858.0 | 0.29 |
| Healthy | 0.15 | 0.61 | 1069.4 | 0.08 |
| Healthy | 0.25 | 0.63 | 748.7 | 0.37 |
| Healthy | 0.23 | 0.82 | 631.8 | 0.26 |
| Healthy | 0.24 | 1 | 1264.0 | 0.11 |
| Healthy | 0.32 | 0.91 | 1082.4 | 0.32 |
| Healthy | 0.31 | 1.74 | 1401.9 | 0.26 |
| Healthy | 0.34 | 1.07 | 1416.1 | 0.36 |
| Healthy | 0.3 | 1.05 | 1220.7 | 0.12 |
| Healthy | 0.53 | 1.75 | 990.3 | 0.09 |
| Healthy | 0.18 | 0.66 | 1017.8 | 0.19 |
| Healthy | 0.12 | 0.33 | 425.9 | 0.33 |
| Healthy | 0.22 | 0.77 | 970.0 | 0.13 |
| Healthy | 0.21 | 0.68 | 707.9 | 0.23 |
| Healthy | 0.45 | 1.28 | 2267.1 | 0.56 |
| Healthy | 0.43 | 1.11 | 1669.1 | 0.25 |
| Healthy | 0.36 | 1.04 | 1117.7 | 0.03 |
| Healthy | 0.03 | 0.08 | 60.6 | 0.12 |
| Healthy | 0.16 | 0.48 | 683.4 | 0.08 |
| Healthy | 0.32 | 1.22 | 524.6 | 0.19 |
| Healthy | 0.65 | 2.01 | 2602.7 | 0.16 |
| Healthy | 0.18 | 0.74 | 878.0 | 0.13 |
| Healthy | 0.43 | 1.4 | 560.9 | 0.18 |
| Healthy | 0.08 | 0.31 | 580.5 | 0.04 |
| Healthy | 0.19 | 0.62 | 485.9 | 0.16 |
| Healthy | 0.44 | 1.29 | 822.7 | 0.14 |
| Healthy | 0.86 | 2.14 | 540.0 | 0.18 |
| Healthy | 0.25 | 0.82 | 2632.6 | 0.1 |
| Healthy | 0.28 | 0.71 | 1594.3 | 0.33 |
| Healthy | 0.45 | 2.37 | 1502.7 | 0.24 |
| Healthy | 0.14 | 0.43 | 699.3 | 0.44 |
| Healthy | 0.37 | 0.77 | 1030.0 | 0.05 |
| Healthy | 0.84 | 1.86 | 886.6 | 0.37 |
| Healthy | 0.17 | 1.4 | 1088.1 | 0.2 |
| Healthy | 0.35 | 1.19 | 661.0 | 0.38 |
| Healthy | 0.3 | 1.09 | 1236.1 | 0.05 |
| Healthy | 0.88 | 3.64 | 895.4 | 0.1 |
| Healthy | 0.43 | 2.02 | 742.3 | 0.26 |
| Healthy | 0.53 | 2.28 | 1246.9 | 0.26 |
| Healthy | 0.32 | 0.9 | 493.3 | 0.13 |
| Healthy | 0.12 | 0.5 | 1042.5 | 0.09 |
| Healthy | 0.18 | 0.48 | 540.4 | 0.1 |
| Healthy | 0.46 | 1.66 | 822.1 | 0.23 |
| Healthy | 0.36 | 1.12 | 1077.2 | 0.1 |
| Healthy | 0.08 | 0.36 | 1023.5 | 0.26 |
| Healthy | 0.44 | 1.07 | 1776.5 | 0.09 |
| Healthy | 0.17 | 0.51 | 616.9 | 0.34 |
| Healthy | 0.36 | 1.41 | 1283.0 | 0.12 |
| Healthy | 0.26 | 0.85 | 659.8 | 0.61 |
| Healthy | 0.61 | 1.02 | 1451.6 | 0.13 |
| Healthy | 0.25 | 0.61 | 532.5 | 0.27 |
| Healthy | 0.54 | 1.52 | 887.1 | 0.07 |
| Healthy | 0.12 | 0.51 | 762.1 | 0.19 |
| Healthy | 0.34 | 0.92 | 1041.7 | 0.12 |
| Healthy | 0.18 | 0.87 | 783.2 | 0.15 |
| Healthy | 0.46 | 1.98 | 642.3 | 0.11 |
| Healthy | 0.78 | 1.52 | 304.9 | 0.09 |
| Healthy | 0.31 | 1.08 | 2591.2 | 0.24 |
| Healthy | 0.57 | 2.01 | 1069.4 | 0.29 |
| Healthy | 0.39 | 1.5 | 692.1 | 0.03 |
| Healthy | 0.28 | 1.42 | 1196.1 | 0.34 |
| Healthy | 0.29 | 1.11 | 1188.4 | 0.15 |
| Healthy | 0.68 | 1.39 | 998.0 | 0.13 |
| Healthy | 0.51 | 1.89 | 1227.2 | 0.1 |
| Healthy | 0.43 | 1.47 | 847.1 | 0.18 |
| Healthy | 0.17 | 0.51 | 408.3 | 0.17 |
| Healthy | 0.82 | 1.73 | 724.0 | 0.27 |
| Healthy | 0.24 | 1.11 | 2485.3 | 0.15 |
| Healthy | 0.86 | 1.87 | 491.6 | 0.08 |
|  |  |  |  |  |
